# The relation of radiological tumor volume response to histological response and outcome in patients with localized Ewing Sarcoma

**DOI:** 10.1002/cam4.2002

**Published:** 2019-02-21

**Authors:** Lianne M. Haveman, Andreas Ranft, Henk vd Berg, Anne Smets, Jarmila Kruseova, Ruth Ladenstein, Benedicte Brichard, Michael Paulussen, Thomas Kuehne, Heribert Juergens, Stephanie Klco‐Brosius, Uta Dirksen, Johannes H.M. Merks

**Affiliations:** ^1^ Emma Children's Hospital Department of Pediatric Oncology Academic Medical Center Amsterdam The Netherlands; ^2^ Prinses Maxima Center for Pediatric Oncology Utrecht The Netherlands; ^3^ Department of Pediatric Hematology and Oncology University of Essen Essen Germany; ^4^ Coordinating Center for Clinical Trials Muenster Germany; ^5^ Department of Radiology Academic Medical Center Amsterdam The Netherlands; ^6^ Department of Pediatric Oncology University Hospital Motol Prague Czech Republic; ^7^ Children's Cancer Research Institute Vienna Austria; ^8^ Saint Luc University Hospital Department of Pediatric Hematology and Oncology University of Louvain Datteln Belgium; ^9^ Witten/Herdecke University Vestische Kinder‐ und Jugendklinik, Datteln Germany; ^10^ Department of Pediatric Oncology and Haematology University Children Hospital Basel Switzerland; ^11^ Department of Pediatric Hematology and Oncology University Children's Hospital Muenster Germany

**Keywords:** Ewing Sarcoma, histology, MRI, outcome, radiological response, tumor volume response

## Abstract

**Background:**

Magnetic resonance imaging (MRI) is the modality of choice for local staging and response evaluation of Ewing sarcoma (EwS). Aim of this study was to determine the relevance of tumor volume response (TVR) in relation to histological response (HisRes) and survival, in order to evaluate if early modification of chemotherapy might be indicated in patients with inadequate TVR.

**Methods:**

Three dimensional (3D)‐tumor volume data at diagnosis, during early induction phase (1‐3 courses of chemotherapy; n = 195) and/or late induction phase (4‐6 courses; n = 175) from 241 localized patients were retrospectively analyzed. A distinction was made between adequate response (reduction ≥67%) and inadequate response (reduction <67% or progression). Correlations between TVR, HisRes, event free survival (EFS), and overall survival (OS) were analyzed using chi‐square tests, log‐rank tests, and the Cox‐regression model.

**Results:**

Early adequate TVR, noted in 41% of patients, did not correlate with EFS (*P* = 0.92) or OS (*P* = 0.38). During late induction phase 62% of patients showed an adequate TVR. EFS for patients with late adequate TVR was better (78%) than for those with inadequate late TVR (61%) (*P* = 0.01); OS was 80% and 69% (*P* = 0.26), respectively. No correlation was found between TVR and HisRes. Multivariate analysis showed that poor HisRes, pelvic location and late inadequate TVR were associated with poor outcome.

**Conclusions:**

Early inadequate TVR does not predict adverse outcome; therefore, changing the treatment to second line chemotherapy is not indicated in case of inadequate early TVR. Late adequate TVR and good HisRes correlate with better EFS; patients with late inadequate TVR might benefit from augmented therapy.

## 1 INTRODUCTION

Ewing sarcoma (EwS) is the second most common malignant bone tumor in childhood, predominantly affecting children and young adults.^1^ Histologically, small blue round cells are noted. Up to 95% of patients harbor the characteristic chromosomal 11q22q translocation.^2^

The backbone of EwS treatment is induction chemotherapy that treats micrometastatic disease and reduces tumor volume to facilitate surgical procedures. Induction chemotherapy is followed by local therapy (surgery and/or radiotherapy) and maintenance chemotherapy. This multimodal treatment has greatly improved survival rate for EwS patients. Overall survival (OS) for patients with localized disease is approximately 70%‐75%; however, still below 30% for patients with metastatic disease.^1^, ^3^

In the daily clinic, there is a need for prognostic factors that give an early indication whether response of the tumor to induction chemotherapy is adequate or whether chemotherapy should be adjusted in case of inadequate response. Histological response (HisRes) has been identified as a prognostic factor for outcome;^4^, ^5^ however, histological response is known late in the treatment, after induction chemotherapy and after local therapy has been performed, and cannot be identified in patients only receiving radiotherapy.

At diagnosis of EwS there is often major soft tissue involvement, which substantially contributes to the total tumor volume. After induction chemotherapy, there is usually a decrease in the soft tissue component, whereas the bony component shows little regression, probably due to the slow regression of the osteoid matrix.^6^, ^7^, ^8^ Based on the superior assessment of intramedullary and soft tissue extension, magnetic resonance imaging (MRI) is considered to be the modality of choice for local staging and response evaluation.^6^, ^9^, ^10^ Early modification of chemotherapy or an intensification of radiotherapy dosage could be potential options for patients with inadequate radiological response noted during early or late induction chemotherapy. Modification could exist of changing to second line agents or the addition of new, often targeted, agents.

Since HisRes has been identified as a prognostic factor, image‐based response might well correlate with HisRes.^11^, ^12^, ^13^, ^14^, ^15^, ^16^ Previously published reports on imaging based tumor response cited only limited numbers of patients and focused more on response during late induction chemotherapy.

This study aims to evaluate the prognostic value of the radiological tumor response with MRI during early and late induction chemotherapy with respect to survival and HisRes in a large cohort of patients with localized EwS. Patients were uniformly treated and included in the international EURO‐E.W.I.N.G.99 (EE99) trial with adequately long follow‐up.^17^

## 2 METHODS

### 2.1 Study population

Data from consecutive Ewing sarcoma patients included in the EE99 trial between September 1999 and September 2009 were analyzed.^17^ Of these only patients with localized disease and all patients whose data contained 3D tumor volume measurements both at diagnosis and at least once during induction chemotherapy were included in the study. Patients who had received local therapy earlier than per protocol were excluded from the analysis. Primary EwS was confirmed by pathology and molecular diagnostics in all patients. All patients and/or legal guardians had given informed consent prior to study entry.

### 2.2 Treatment

The EE99 protocol mandated 6 courses of induction chemotherapy consisting of vincristine, ifosfamide, doxorubicin, and etoposide (VIDE). Following local therapy, a risk‐adapted chemotherapy was administered. Protocol treatment details have been previously published.^17^

### 2.3 Definitions

#### 2.3.1 Tumor volume

Three‐dimensional tumor volume was calculated from MRI measurements (anteroposterior dimension x transverse x craniocaudal dimension). In the EE99 study a correction factor (F) was applied to elliptically shaped tumors (F = π/6 = 0.52) and to cylindrically shaped tumors (F = π/4 = 0.785). For all other tumor shapes, a correction factor of 0.63 (F = π/5) was applied. The same correction factor used for the MRI at diagnosis was used for subsequent MRIs. Changes in tumor volume were expressed as percentage involution. Adequate response (reduction ≥67%) and inadequate response (reduction <67% or progressive disease) were delineated in line with cutoff values for partial response (PR) according to the Response Evaluation Criteria in Solid Tumors (RECIST) response assessment.^18^, ^19^

#### 2.3.2 Histological response

Following induction chemotherapy, local therapy was performed. In patients who underwent surgery, the grade of histopathological response, that is, the percentage of viable tumor cells in the specimen, was determined according to the Salzer‐Kuntschik classification.^20^ A good response was defined as having less than 10% viable tumor cells (grade 1: no vital cells, grade 2: 1%‐4% vital cells, grade 3: 5%‐9% vital cells). A poor response was defined as having more than 10% viable tumor cells (grade 4: 10%‐50% cells, grade 5/6: >50% vital cells).

#### 2.3.3 Survival

EFS time was defined as the interval between the first day of the first VIDE course and the date of first event or death. In the absence of events, patients were censored on the date of their most recent consultation. An event was defined as progressive disease, relapsed disease (local or metastatic), secondary malignancy, or death from any cause. OS was defined as the time from the first day of the first VIDE course until death from any cause. Patients with progressive disease (PD) at time of TVR assessment were excluded from analysis, as PD on therapy is known to be associated with poor outcome.

### 2.4 Statistical analyses

Statistical analyses were performed using SPSS statistics 22 (IBM Corporation, Armonk, NY) and SAS 9.2 (SAS Institute, Cary, NC) software packages. EFS and OS were calculated using the Kaplan–Meier method. A log‐rank test was performed to determine statistical differences in the distribution of survival. Univariate comparisons between groups of patients and statistical significance were accomplished using log‐rank and chi‐square tests. Multivariate test procedures applied Cox and logistic regression analyses. The significance level was set at *P* < 0.05 (two‐sided). Missing data were handled using list wise deletion.

## 3 RESULTS

**Figure 1:**
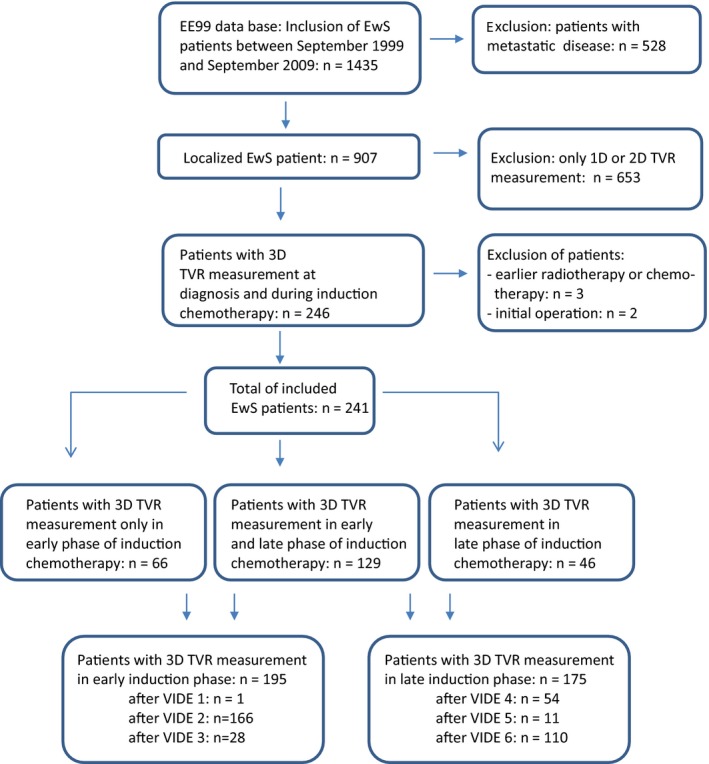
Flow diagram of included patients. EwS, Ewing sarcoma, EE99, Euro‐Ewing 99 trial, TVR, tumor volume response

From in total 1435 patients included in the EE99 trial, 907 patients had localized EwS. In 246 of the 907 patients the 3D tumor volume was calculated from MRI measurements taken at diagnosis and at least once during induction chemotherapy. Two patients who had received local or systemic therapy earlier than per protocol and 3 patients who had received initial surgery were excluded from analysis, so ultimately 241 patients were included in the study (Figure 1). Table 1 shows the clinical baseline characteristics of these patients; these characteristics are consistent with previously published reports;^21^, ^22^ therefore, this study can be considered representative for the EE99 study cohort.

**Table 1:** Characteristics of included EwS patients (n = 241)

Patient characteristics	Number (Total n = 241)	Percentage (%)
Age in years (median (range))		
15.33 (0.42‐56 y)		
Gender		
Male	149	61.8
Female	92	38.2
Tumor volume at diagnosis (median (range))		
161 mL (0.7‐2717 mL)		
< 200 mL	136	56.4
>200 mL	105	43.6
Tumor form at diagnosis		
Elliptic	151	62.7
Cylindrical	38	15.8
Unknown/missing	52	21.5
Localization		
Pelvis	52	21.6
Abdomen	4	1.7
Spine	5	2.0
Thorax	55	22.8
Head and neck	12	5.0
Upper extremity	21	8.7
Lower extremity	92	38.2
Soft tumor component		
Bone with or without soft tissue	215	89.2
Extra‐osseous	25	10.4
Missing	1	0.4
Histopathology		
Undifferentiated EwS	135	56.0
Neuro‐differentiated EwS/PNET	78	32.4
Large cell EwS	3	1.2
Other EwS	17	7.1
Missing	8	3.3
Local therapy		
Surgery alone	123	51.0
Surgery and radiotherapy	95	39.4
Radiotherapy alone	16	6.6
No local therapy	4	1.7
Missing	3	1.2
Surgical resection	Total n = 220	
Radical resection	164	74.5
Marginal resection	39	17.7
Intralesional resection	12	5.5
Missing	5	2.3
Histological response	Total n = 220	
Good: n = 151		
Grade 1: no vital cells	100	45.5
Grade 2: 1%‐4% vital cells	11	5.0
Grade 3: 5%‐9% vital cells	39	17.7
Grade missing	1	0.5
Poor: n = 65		
Grade 4: 10%‐50% vital cells	41	18.6
Grade 5/6: >50% vital cells	23	10.5
Grade missing	1	0.5
Missing	4	1.7

In 129 of 241 included patients, 3D‐tumor volume measurement was performed at diagnosis, during early induction (course 1‐3) and during late induction phases (course 4‐6). If more than one MRI was performed during the early and/or late induction phase, only the data from the latest MRI were used. In 66 patients, an MRI was performed at diagnosis and only during early induction phase; in 46 patients, an MRI was done at diagnosis and only during late induction phase. For patients whose response was not measured with an MRI during the early induction phase (n = 46) or late induction phase (n = 66) other imaging modalities (often CT‐scan or ultrasound) were used for response assessment at these time points; these assessments were not reported in 3D. Ultimately, TVR was determined in 195 patients during early induction phase, and in 175 patients during late induction phase (Figure 1).

In patients with early TVR measurement (n = 193; excluding 2 patients with PD) median tumor reduction was 62%. In 80/193 patients (=41%), an adequate TVR was observed (median reduction = 83%). In the group with inadequate TVR (115/193 patients = 59%) the median reduction was 41%. During late induction phase the median reduction compared to diagnosis was 77% (n = 172; excluding 3 patients with PD). Adequate TVR was observed in 108/172 patients (=62%; median reduction 88%). In the 67/172 patients with an inadequate TVR the median reduction was 40%.

Of the 129 patients who had MRIs during both early and late induction phases, 4 patients showed progressive disease at time of late response assessment compared to their early response assessment. Overall, progression was observed in 9/241 patients (3.7%) during induction chemotherapy.

### 3.1 Combined evaluation of radiological and histological response

Local resection was performed in 220/241 patients (91%); 151 of these 220 patients (70%) showed a good HisRes (see also Table 1). Table 2 illustrates the TVR during early and late induction chemotherapy in relation with the HisRes determined in the patient group who underwent local resection. In the patients with early TVR measurement, 178/195 patients (91%) underwent local resection; however, histological data from 3/178 patients were missing; 122/175 (70%) of the remaining 175 patients showed a good HisRes. Of the 175 patients with TVR measurement during late induction phase, 161/175 (91%) underwent local resection; HisRes of 1 patient was missing. 111/160 patients (69%) showed a good HisRes. No correlation could be found between either early TVR (*P* = 0.92) or late TVR (*P* = 0.63) and histological response.

**Table 2:** Tumor volume response (TVR) in patients who underwent surgical resection (n = 220) and correlation with histological response and TVR and relation to 3‐year Event Free Survival (EFS) and Overall Survival (OS) during early and late induction chemotherapy

Histological response and relation with TVR				TVR and relation with outcome:3 years EFS and OS (SE)			
Patients who underwent surgery(n = 220)	Adequate TVR(% of total)	Inadequate TVR(% of total)	*P*	Total group of patients(n = 241)	Adequate TVR(% of total)	Inadequate TVR(% of total)	*P*
Early TVR measurement (n = 175)	n = 75 (43%)good HR: n = 53 (71%) poor HR:n = 22 (29%)	n = 100 (57%) good HR: n = 69 (69%) poor HR: n = 31 (31%)	*P* = 0.92	Early TVR measurement (n = 194)	n = 79 (41%) EFS 0.74 (SE 0.05)OS 0.74 (SE 0.05)	n = 115 (59%) EFS 0.72 (SE 0.05) OS 0.82 (SE 0.05)	*P* = 0.90 *P* = 0.37
Late TVR measurement (n = 160)	n = 100 (62.5%) good HR: n = 68 (68%) poor HR: n = 32 (32%)	n = 60 (37.5%) good HR: n = 43 (72%) poor HR:n = 17 (28%)	*P* = 0.63	Late TVR measurement (n = 174)	n = 107 (61.5%) EFS 0.78 (SE 0.04) OS 0.8 (SE 0.04)	n = 67 (38.5%) EFS 0.61 (SE 0.06) OS 0.69 (SE 0.04)	*P* = 0.013^a^ *P* = 0.26

HR, histological response.

a Statistically significant.

### 3.2 Factors related to outcome

#### 3.2.1 Radiological response and survival

Median follow‐up was 4.49 years (range 0.12‐13.2). Overall, 3‐year EFS was 0.71 (SE = 0.03) and OS 0.77 (SE = 0.03). This is in line with survival data previously reported from localized EwS patients in the EE99 trial.^21^

#### 3.2.2 Early TVR measurement and survival

**Figure 2:**
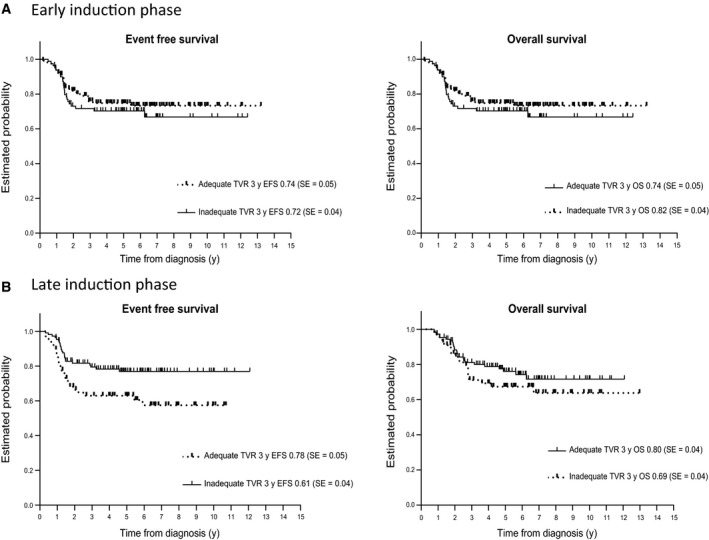
EFS (left) and OS (right) of patients with adequate and inadequate radiological response during (A) the early induction phase of chemotherapy (B) the late induction phase of chemotherapy

For the patient group with early TVR measurements available, survival data were known from 194/195 patients. In 79/194 (41%) of the patients, an adequate TVR was observed and 3‐year EFS was 0.74 (SE 0.05). In patients with early inadequate response, EFS was 0.72 (SE 0.04) (*P* = 0.90). Three‐year OS was 0.74 (SE 0.05) and 0.82 (SE 0.04), respectively (*P* = 0.37) (Table 2; Figure 2A).

#### 3.2.3 Late TVR and survival

In the patient group with late TVR measurements available survival data from 174/175 patients were known. In 61.5% of these patients an adequate TVR was observed; the associated 3‐year EFS was 0.78 (SE 0.04) in contrast to 0.61 (SE 0.06) for patients with inadequate TVR (*P* = 0.013). Three‐year OS rate for patients with adequate TVR was 0.80 (SE 0.04) compared to 0.69 (SE 0.04) for patients with inadequate TVR (*P* = 0.26) (Table 2; Figure 2B).

### 3.3 Histological response and survival

**Figure 3:**
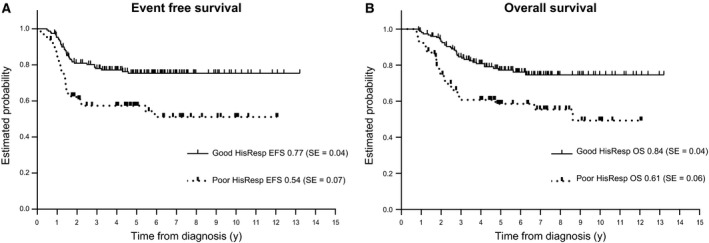
EFS (A) and OS (B) of patients who underwent local surgery (n = 220) with adequate versus inadequate histological response

A good HisRes was observed in 151/216 patients (70%); associated 3‐year EFS was 0.77 (SE 0.04), whereas EFS was 0.54 (SE 0.07) in patients with poor HisRes (*P* = 0.001). Three‐year OS was 0.84 (SE 0.03) and 0.61 (SE 0.06), respectively (*P* = 0.001) (Figure 3).

### 3.4 Other factors associated with outcome

Fifty‐six per cent of patients had a tumor volume <200 mL; associated 3‐year EFS was 0.78 (SE 0.04); significantly better (*P* = 0.005) than EFS for 105 patients with an initial tumor volume ≥200 mL (EFS 0.62; SE 0.05). OS was 0.80 (SE 0.04) in patients with an initial tumor volume <200 mL compared to 0.72 (SE 0.05) for patients with a tumor volume >200 mL (*P* = 0.007).

No statistical difference (*P* = 0.66) was detected in EFS and OS between patients with osseous EwS (EFS 0.73; (SE 0.03); OS 0.77 (SE 0.03)) and extra‐osseous EwS (EFS 0.64; [SE 0.10]; OS 0.64 [SE 0.1] [*P* = 0.33]).

Three‐year EFS was 0.59 (SE 0.07) for patients with pelvic lesions. This was lower (*P* = 0.029) than EFS for EwS patients with other locations (EFS 0.73; SE 0.03). OS was 0.66 (SE 0.07) and 0.80 (SE 0.03), respectively (*P* = 0.04). Patients <15 years of age had higher 3‐year EFS than patients ≥15 years: 0.77 (SE 0.04) compared to 0.64 (SE 0.05; *P* = 0.04). Three‐year OS was 0.83 (SE 0.04) and 0.70 (SE 0.04), respectively (*P* = 0.007). Three‐year EFS was 0.66 (SE 0.04) for males and 0.79 (SE 0.04) for females (*P* = 0.03). OS was 0.74 (SE 0.04) and 0.81 (SE 0.04), respectively (*P* = 0.26).

Surgery was radical in 164/214 patients (77%); marginal in 39/214 (18%) and intralesional in 11 patients (5%). Three‐year EFS was 0.73 (SE 0.04), 0.63 (SE 0.78), and 0.72 (SE 0.14), respectively (*P* = 0.65). Three‐year OS was 0.80 (SE 0.03), 0.66 (SE 0.08), and 0.70 (SE 0.14), respectively (*P* = 0.21).

### 3.5 Multivariate analysis

Based on the factors associated with a significantly inferior 3‐year EFS in univariate analysis, a multivariate analysis was performed. Detailed results are shown in Table 3. A poor histological response after induction chemotherapy, a late 3D‐TVR and pelvic site were independent predictors for inferior EFS. For OS, poor HisRes and pelvic site were independent predictors (*P* < 0.05; late inadequate TVR: *P* = 0.10).

**Table 3:** Multivariate analysis by Cox regression of factors associated with disease outcome in patients with localized EwS during late induction phase (n = 175)

Covariate	EFS			OS		
	HR	95% CI	*P*	HR	95% CI	*P*
Age > 15 years	1.04	0.56‐1.91	0.91	1.36	0.72‐2.58	0.35
Sex	0.62	0.32‐1.19	0.15	0.89	0.47‐1.70	0.73
Initial tumor volume > 200 mL	1.73	0.91‐3.30	0.10	1.58	0.81‐3.11	0.18
Poor histological response (>10% viable cells)	2.28	1.25‐4.15	0.007^a^	2.23	1.20‐4.15	0.011^a^
Late inadequate tumor volume response (3D)	2.26	1.23‐4.15	0.008^a^	1.69	0.89‐3.2	0.10
Pelvic location	2.80	1.49‐5.40	0.001^a^	2.44	2.44‐1.25	0.01^a^

CI, confidence interval; HR, Hazard ratio.

a Difference *P* < 0.05.

## 4 DISCUSSION

Radiological response to chemotherapy as a prognostic factor for outcome has been described in several studies. In this study, we analyzed three‐dimensional tumor volume response in a large series of patients uniformly treated according to the Euro Ewing 1999 protocol to evaluate if early modification of chemotherapy could be an option in patients with an early inadequate TVR. However, we showed that early TVR is not related to prognosis nor to HisRes. Therefore, modification of chemotherapy in response to inadequate early TVR may not be justifiable. However, we did find that late inadequate TVR, poor HisRes and pelvic tumor location are independently associated with poor prognosis.

Studies concerning radiological TVR and correlation with survival were performed in small patient cohorts and showed inconsistent results.^11^, ^12^, ^13^, ^14^, ^15^, ^16^ In this respect, the large number of EwS patients included in our study is novel and adds to the available evidence. Limitations of this study include its retrospective nature; volume measurement was not centrally reviewed and a limited sample size of included patients when compared to the total number of patients included in the Euro Ewing 99 trial.

TVR may not adequately reflect the complex histological changes resulting from response to chemotherapy. Therefore, besides TVR, also the residual extramedullary tumor volume, calcification of residual tumor in soft tissue and medullary cavity, T2 signal variations, diffusion weighted imaging, and fludeoxyglucose positron emission tomography have been investigated as potential markers for response.^6^, ^7^, ^23^, ^24^, ^25^, ^26^, ^27^, ^28^, ^29^ Taking also (some of) these factors into account, a better prediction of tumor response in early induction might be possible. There is some debate as to which type of measurement (3D/2D/1D) is most valuable in assessing radiological response in solid tumors. Three‐dimensional tumor measurement has been shown to be more sensitive and accurate than 1D or 2D assessment in patients with other solid tumors,^26^, ^30^ whereas another study could not show a difference between 3D and 1D RECIST in rhabdomyosarcoma.^18^ Three‐dimensional TVR sensitivity might be more evident for tumors in which the axial tumor area changes more than the longitudinal dimension, which is commonly seen in bone tumors.^31^ Aghighi et al compared 1D, 2D, and 3D tumor measurements with clinical outcomes for EwS patients. Three‐dimensional tumor measurement was shown to be superior, and a higher correlation with clinical outcome was described.^16^

In our study, 41% of patients achieved adequate TVR during the early phase of induction therapy and 62% during late induction phase. These percentages are slightly better than the TVR reported by Pan et al*,* 13 who examined 66 localized and metastatic EwS patients prior to surgery. Nearly 40% of patients achieved at least partial remission (PR) according to RECIST criteria. An important question in daily clinical practice is whether early modification of chemotherapy is warranted in patients with early inadequate radiological tumor volume response. None of the previously published studies focused on TVR during early induction phase, where results of our study now show that TVR during early induction phase does not correlate with survival, and therefore modification of chemotherapeutic regimen should not be based on TVR during early induction phase. However, 3‐year EFS was higher for patients with adequate TVR measured late during induction chemotherapy, compared to patients with inadequate response at this time point. Furthermore, adequate TVR during either early or late chemotherapy induction phases was not a predictive marker for HisRes. This is in agreement with the observations of Pan et al.^13^ Abudu et al found a significant relationship between necrosis and 3D‐TVR;^12^ however, this study included only 50 EwS patients, both localized and metastatic, and used MRI or CT‐scan to determine TVR.

Similar to other studies,^4^, ^11^, ^12^, ^32^ we found that poor HisRes to induction chemotherapy correlated with poor outcome. In addition to poor HisRes and late inadequate TVR, initial tumor volume >200 ml, age older than 15 years, pelvic location and male gender all appeared to be bad prognostic factors as determined with univariate analysis. These are all well‐known unfavorable prognostic factors.^2^, ^3^, ^21^, ^22^, ^32^ Multivariate analysis revealed that poor HisRes, pelvic location and late inadequate TVR were independently associated with poor prognosis. Based on EFS, patients with late inadequate TVR or poor HisRes on standard therapy, as a result of intrinsic tumor resistance to applied chemotherapeutic agents, could benefit from a more augmented therapy. For patients who receive radiotherapy only and therefore lack histological response as prognostic marker, late TVR may help to adequately stratify those patients for subsequent chemotherapy.

In conclusion, based on survival data, modifying chemotherapy based on inadequate early TVR may not be justifiable; however, patients with late‐phase inadequate TVR might benefit from augmented treatment.

## CONFLICT OF INTEREST

The funding made this study financially possible, and had no role in study design or interpretation. There were no conflicts of interest disclosures from any authors.

## References

[1] Potratz J , Jürgens H , Craft A , Dirksen U . Ewing sarcoma: biology‐based therapeutic perspectives. Pediatr Hematol Oncol. 2012;29(1):12‐27. 22304007 10.3109/08880018.2011.627582

[2] Ladenstein R , Pötschger U , Le Deley MC , et al. Primary disseminated multifocal Ewing sarcoma: results of the Euro‐EWING 99 trial. J Clin Oncol. 2010;28(20):3284‐3291. 20547982 10.1200/JCO.2009.22.9864

[3] Cotterill SJ , Ahrens S , Paulussen M , et al. Prognostic factors in Ewing's tumor of bone: analysis of 975 patients from the European intergroup Cooperative Ewing's Sarcoma Study Group. J Clin Oncol. 2000;18(17):3108‐3114. 10963639 10.1200/JCO.2000.18.17.3108

[4] Picci P , Rougraff BT , Bacci G , et al. Prognostic significance of histopathologic response to chemotherapy in nonmetastatic Ewing's sarcoma of the extremities. J Clin Oncol. 1993;11(9):1763‐1769. 8355043 10.1200/JCO.1993.11.9.1763

[5] Sauer R , Jürgens H , Burgers JM , Dunst J , Hawlicek R , Michaelis J . Prognostic factors in the treatment of Ewing's sarcoma. The Ewing's Sarcoma Study Group of the German Society of Paediatric Oncology CESS 81. Radiother Oncol 1987;10(2):101‐110. 3423301 10.1016/s0167-8140(87)80052-x

[6] Brisse H , Ollivier L , Edeline V , et al. Imaging of malignant tumours of the long bones in children: monitoring response to neoadjuvant chemotherapy and preoperative assessment. Pediatr Radiol. 2004;34(8):595‐605. 15103428 10.1007/s00247-004-1192-x

[7] Lemmi MA , Fletcher BD , Marina NM , et al. Use of MR imaging to assess results of chemotherapy for Ewing sarcoma. AJR Am J Roentgenol. 1990;155(2):343‐346. 2115265 10.2214/ajr.155.2.2115265

[8] van der Woude HJ , Bloem JL , Hogendoorn PC . Preoperative evaluation and monitoring chemotherapy in patients with high‐grade osteogenic and Ewing's sarcoma: review of current imaging modalities. Skeletal Radiol. 1998;27(2):57‐71. 9526770 10.1007/s002560050339

[9] Bloem JL , Taminiau AH , Eulderink F , Hermans J , Pauwels EK . Radiologic staging of primary bone sarcoma: MR imaging, scintigraphy, angiography, and CT correlated with pathologic examination. Radiology. 1988;169(3):805‐810. 3055041 10.1148/radiology.169.3.3055041

[10] Leung JC , Dalinka MK . Magnetic resonance imaging in primary bone tumors. Semin Roentgenol. 2000;35(3):297‐305. 10939131 10.1053/sroe.2000.7340

[11] van der Woude HJ , Bloem JL , Holscher HC , et al. Monitoring the effect of chemotherapy in Ewing's sarcoma of bone with MR imaging. Skeletal Radiol. 1994;23(7):493‐500. 7824974 10.1007/BF00223076

[12] Abudu A , Davies AM , Pynsent PB , et al. Tumour volume as a predictor of necrosis after chemotherapy in Ewing's sarcoma. J Bone Joint Surg Br 1999;81(2):317‐322. 10204943 10.1302/0301-620x.81b2.8979

[13] Pan HY , Morani A , Wang WL , et al. Prognostic factors and patterns of relapse in ewing sarcoma patients treated with chemotherapy and r0 resection.Int J Radiat Oncol Biol Phys 2015;1;92(2):349‐357. 10.1016/j.ijrobp.2015.01.022 PMC4431926 25772182

[14] Golfieri R , Baddeley H , Pringle JS , Leung AWL , Greco A , Souhami R . MRI in primary bone tumours: therapeutic implications. Eur J Radiol. 1991;12:201‐207. 1855513 10.1016/0720-048x(91)90073-5

[15] Miller SL , Hoffer FA , Reddick WE , et al. Tumor volume or dynamic contrast‐enhanced MRI for prediction of clinical outcome of Ewing sarcoma family of tumors. Pediatr Radiol. 2001;31(7):518‐523. 11486808 10.1007/s002470100481

[16] Aghighi M , Boe J , Rosenberg J , et al. Three‐dimensional radiologic assessment of chemotherapy response in Ewing Sarcoma can be used to predict clinical outcome. Radiology. 2016;280(3):905‐915. 26982677 10.1148/radiol.2016151301 PMC5006736

[17] Juergens C , Weston C , Lewis I , et al. Safety assessment of intensive induction with vincristine, ifosfamide, doxorubicin, and etoposide (VIDE) in the treatment of Ewing tumors in the EURO‐E.W.I.N.G. 99 clinical trial. Pediatr Blood Cancer. 2006;47(1):22‐29. 16572419 10.1002/pbc.20820

[18] Schoot RA , McHugh K , van Rijn RR , et al. Response assessment in pediatric rhabdomyosarcoma: can response evaluation criteria in solid tumors replace three‐dimensional volume assessments? Radiology. 2013;269(3):870‐878. 23985275 10.1148/radiol.13122607

[19] Gehan EA , Tefft MC . Will there be resistance to the RECIST (Response Evaluation Criteria in Solid Tumors)? J Natl Cancer Inst. 2000;92(3):179‐181. 10655425 10.1093/jnci/92.3.179

[20] Salzer‐Kuntschik M , Delling G , Beron G , Sigmund R . Morphological grades of regression in osteosarcoma after polychemotherapy‐study COSS 80. J Cancer Res Clin Oncol. 1983;106(Suppl):21‐24. 10.1007/BF00625047 PMC12253038 6577010

[21] Le Deley M , Paulussen M , Lewis I , et al. Cyclophosphamide compared with Ifosfamide in consolidation treatment of standard‐risk Ewing Sarcoma: results of the randomized noninferiority Euro‐EWING99‐R1 Trial. J Clin Oncol. 2014;32:2440‐2448. 24982464 10.1200/JCO.2013.54.4833

[22] Grevener K , Haveman LM , Ranft A , et al. Management and outcome of Ewing Sarcoma of the head and neck. Pediatr Blood Cancer. 2016;63:604‐610. 26702872 10.1002/pbc.25830

[23] Bedetti B , Wiebe K , Ranft A , et al. Use of MR imaging to assess results of chemotherapy for Ewing sarcoma. AJR. 1990;155:343‐346. 2115265 10.2214/ajr.155.2.2115265

[24] Mac Vicar AD , Olliff JFC , Pringle J , Ross Pinkerton C , Husband JES . Ewing sarcoma: MR imaging of chemotherapy‐induced changes with histologic correlation. Radiology. 1992;184:859‐864. 1509080 10.1148/radiology.184.3.1509080

[25] Shapeero LG , Vanel D . Imaging evaluation of the response of high grade osteosarcoma and Ewing sarcoma to chemotherapy with emphasis on dynamic contrast‐enhanced magnetic resonance imaging. Semin Musculoskelet Radiol. 2000;4(1):137‐146. 11061698 10.1055/s-2000-6861

[26] Burke M , Anderson JR , Kao SC , et al.; Soft Tissue Sarcoma Committee of the Children's Oncology Group . Assessment of response to induction therapy and its influence on 5‐year failure‐free survival in group III rhabdomyosarcoma: the Intergroup Rhabdomyosarcoma Study‐IV experience–a report from the Soft Tissue Sarcoma Committee of the Children's Oncology Group. J Clin Oncol 2007;25(31):4909‐4913. 17971587 10.1200/JCO.2006.10.4257

[27] Chen L , Liu M , Bao J , et al. The correlation between apparent diffusion coefficient and tumor cellularity in patients: a meta‐analysis. PLoS ONE 2013;8(11):e79008. 24244402 10.1371/journal.pone.0079008 PMC3823989

[28] Bailly C , Leforestier R , Campion L , et al. Prognostic value of FDG‐PET indices for the assessment of histological response to neoadjuvant chemotherapy and outcome in pediatric patients with Ewing sarcoma and osteosarcoma. PLoS ONE. 2017;12(8):e0183841. 28841702 10.1371/journal.pone.0183841 PMC5571925

[29] Degnan AJ , Chung CY , Shah AJ . Quantitative diffusion‐weighted magnetic resonance imaging assessment of chemotherapy treatment response of pediatric osteosarcoma and Ewing sarcoma malignant bone tumors. Clin Imaging. 2018;47:9‐13. 28806574 10.1016/j.clinimag.2017.08.003

[30] Mozley PD , Schwartz LH , Bendtsen C , Zhao B , Petrick N , Buckler AJ . Change in lung tumor volume as a biomarker of treatment response: a critical review of the evidence. Ann Oncol. 2010;21(9):1751‐1755. 20332135 10.1093/annonc/mdq051

[31] Silva FD , Pinheiro L , Cristofano C , de Oliveira Schiavon JL , Lederman HM . Magnetic resonance imaging in pediatric bone tumors. Curr Radiol Rep.. 2014;2(77):2‐11.

[32] Oberlin O , Deley MC , Bui BN , et al. J French Society of Paediatric Oncology . Prognostic factors in localized Ewing's tumours and peripheral neuroectodermal tumours: the third study of the French Society of Paediatric Oncology (EW88 study). Br J Cancer 2001;85(11):1646‐1654. 11742482 10.1054/bjoc.2001.2150 PMC2363978

